# The Effect of Oral Feeding of *Tribulus terrestris* L. on Sex Hormone and Gonadotropin Levels in Addicted Male Rats

**Published:** 2013-03-06

**Authors:** Mohammad Hassan Ghosian Moghaddam, Mohsen Khalili, Maryam Maleki, Mohammad Esmail Ahmad Abadi

**Affiliations:** 1 Department of Biochemistry, Shahed University, Tehran, Iran; 2 Neurophysiology Research Center, Shahed University, Tehran, Iran; 3 Shahed University, Tehran, Iran

**Keywords:** Morphine, *Tribulus terrestris*, FSH, LH, Sex Hormones

## Abstract

**Background:**

Opioids can exert adverse effects on the body. Morphine, an opioid drug, reduces hormone levels and fertility, and causes sexual activity disorders. *Tribulus terrestris* (TT) is a traditional herbal medicine used to enhance sexual activities. This study investigates the possible role of TT on sex hormones and gonadotropins with the intent to show its usefulness in treating fertility disorders in opioid users.

**Materials and Methods:**

In this experimental study, we randomly divided 48 rats into four groups: i. control, ii. TT-treated, iii. addicted and iv. TT-treated addicted. Watersoluble morphine was administrated orally for 21 days to induce addiction, after which the treated groups 2 and 4 received plant-mixed pelleted food (6.25%) orally for four weeks. At the end of the treatment period, the sex hormone and gonadotropin levels of all rats’ sera were determined by radioimmunoassay and Elisa kits. The data obtained were statistically analyzed using the one-way analysis of variance, followed by post-hoc Tukey test. P<0.05 was considered significant.

**Results:**

The addicted group had a significantly lower luteinizing hormone (LH) level than the control group (p<0.027). LH levels increased significantly in the TT-treated addicted group (p<0.031). The testosterone level in the treated addicted group was lower than the treated control group. The addicted group had a significantly low testosterone level (p<0.001). The estrogen level was significantly (p<0.002) lower in the addicted group than in the control group. In addition, there was a significant difference between the treated addicted group and the treated control group (p<0.048). The treated control group had a significant increase in its progesterone level (p<0.002). Overall, except for follicle-stimulating hormone (FSH), morphine reduced most of the gonadotropins and sexual hormones. Whereas TT caused a considerable increase (p<0.05) in the hormones in the treated addicted group, there was only a slight increase in the treated control group.

**Conclusion:**

Oral consumption of TT could markedly antagonize the reduction of sex hormones and gonadotropins (except for FSH) due to morphine addiction.

## Introduction

Recent years have witnessed a dramatic rise in the use of opioid drugs despite the documentation of their numerous adverse effects in the literature. One of these side effects is the negative impact on sex hormone levels, libido, potency, and menorrhea (1-3). A study has shown that 96% of men and 69% of women who receive opioid analgesic drugs for pain management have decreased libido or impotency (4). Also, it has been found that spinal opiate analgesics reduce libido and cause difficulty in achieving or maintaining an erection in men (5).

One of the most commonly used opioid drugs is morphine. In a prospective, uncontrolled, non-randomized study, a group of men with an average pain duration of 11 years used intrathecal morphine for 12 weeks. Most of the patients reported poor libido and erectile difficulty toward the end of the 12-week period; in addition, testosterone and follicle-stimulating hormone (FSH) levels significantly reduced. The intrathecal opioid caused a reduction in hormone levels (6).

Tribulus terrestris (TT), commonly known as caltrop or devil’s eyelashes plays an important role in traditional medicine. Most parts of this plant are used in herbal medicine, for which TT can enhance sex drive and treat urolithiasis, menorrhagia, impotency, rheumatism pains, and premature ejaculation (7-9). Khordadmehr and his colleagues have used TT in an herbal formula, NOFODA, to investigate its effect on infertile males and concluded that it improved both sperm motility and count (10).

In studies conducted on animals, TT is thought to have a luteinizing hormone (LH)-like activity, which can induce corpus luteum formation in female rats (11). LH induces the corpus luteum to secrete progesterone. This results in an increase in progesterone levels as well as some estrogen secretion. Studies have shown that protodioscin, which is found in TT extract, treats mild to moderate erectile dysfunction and increases libido (12, 13).

In light of the increasing fertility disorders in opioid users, the present study investigated the effect of TT on the sex hormones and gonadotropins of addicted male rats.

## Materials and Methods

### Experimental animals

Adult male Wister rats that weighed 200 ± 25 g each (Razi Institute, Iran) were randomly divided into four groups, of 12 animals each: i. control, ii. TT-treated, iii. addicted and iv. TT-treated ad- Ghosian Moghaddam et al. dicted. The rats were housed in groups of three in cages at a temperature of 22-25˚C, on a 12 hour light/12 hour dark schedule and sufficient amounts of food and water.

### Preparation of Tribulus terrestris (TT)

After obtaining TT and verifying its suitability for our study via the Department of Botany at Shahid Beheshti University, we ground and combined the plant with pelleted food at a weight ratio of 6.25%.

### Study protocol

Morphine addiction was induced according to the method of Moini Zanjani et al. (14). Groups 3 and 4 received oral administrations of watersoluble morphine for 21 days. During this 21-day period, the treated groups (2 and 4) also received oral administration of TT plant-mixed pelleted food (6.25%).

The water soluble morphine solution was given in doses of 0.1, 0.2, and 0.3 mg/ml according to the method of Swanston-Flatt et al. (15); each of these doses was administered for 48 hours for the rats to drink and then 0.4 mg/ml was administered for the remaining 15 days. The bitterness of morphine was eliminated by adding 3% sucrose to the solution. In this experiment, the average amount of water consumed, and therefore morphine, by rats was approximately 60-80 mg/ml/day. Addiction was confirmed by injecting a morphine antagonist (naloxone) and observing for withdrawal symptoms.

All techniques and methods were approved by the Ethics Committee of Shahed University of Medical Sciences. The laboratory animals were afforded due care in accordance with the regulations of the Committee for the Purpose of Control and Supervision on Experiments on Animals (CPCSEA).

### Blood sampling

After 21 days of treatment, at treatment termination, blood samples (3-5cc) were obtained from all the rats’ hearts to measure hormone levels. The sera were subsequently separated via centrifuge (Sigma 4-10, USA) at 2000 rpm for 10 minutes and stored at -70˚C in a freezer until hormone analysis.

### Plasma analysis

The plasma concentrations of the sex hormones and gonadotropins were verified by the radioimmunoassay (RIA) method using kits manufactured by Monobind Inc. (USA), as well as ELISA kits (Labsystem, Finland).

### Statistical analysis

Obtained data were expressed as mean ± SEM and statistically analyzed using the one-way analysis of variance (ANOVA), followed by the posthoc Tukey test. p<0.05 was considered statistically significant.

## Results

### FSH analysis

**Fig 1:**
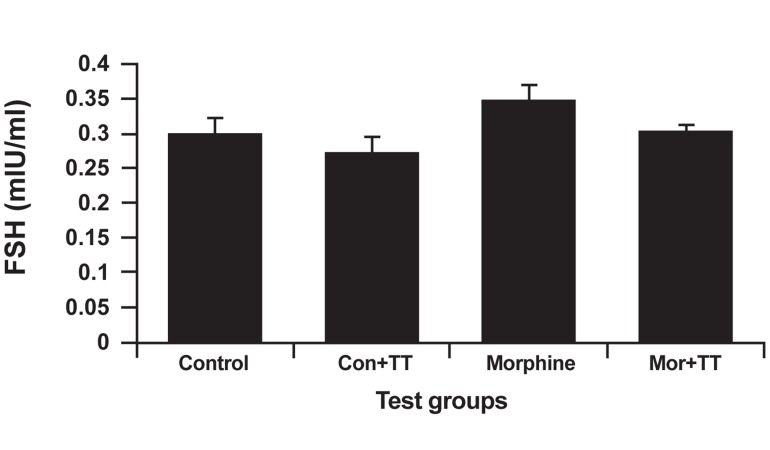
The effect of Tribulus terrestris (TT) on FSH levels in control and addicted groups. Bars depict mean ± SEM of LH.

Our FSH analysis showed that the TT-treated control group had the least amount of FSH (0.271 ± 0.025 mIU/ml), while the morphine-addicted group had the highest FSH level (0.348 ± 0.022 mIU/ml). Morphine solely did not suppress FSH. The TT-treated addicted group had a higher FSH level (0.302 ± 0.01 mIU/ml) than did the TT-treated control group; there was, however, no significant change in any of the groups. Figure 1 illustrates these results.

### LH analysis

**Fig 2:**
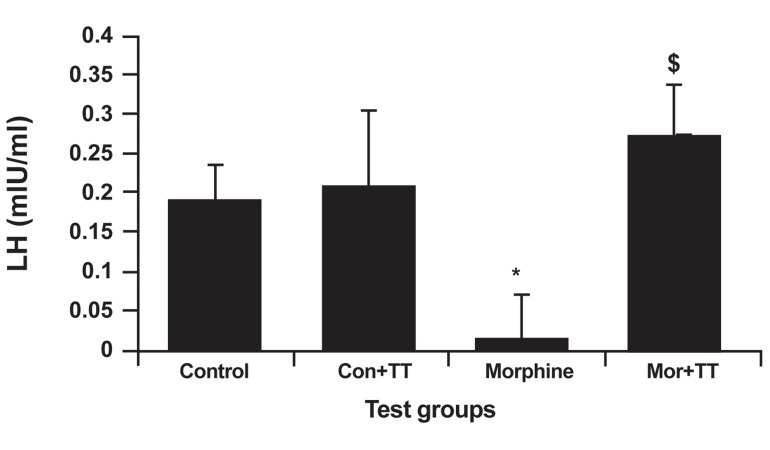
The effect of Tribulus terrestris (TT) on LH levels in control and addicted groups. Bars depict mean ± SEM of LH.* and $; P<0.05 compared between the control and treated control groups.

In LH analysis, the addicted group had decreased LH levels. The highest amount of LH belonged to the treated control group (0.207 ± 0.098 mIU/ml). Figure 2 shows a significantly lower LH level in the addicted group than the control group (0.0125 ± 0.017 mIU/ml), which was due to morphine suppression (p<0.027). The TT-treated addicted group had a significantly higher amount of LH (0.273 ± 0.066 mIU/ml) than did the control group p<0.031.

### Testosterone analysis

**Fig 3:**
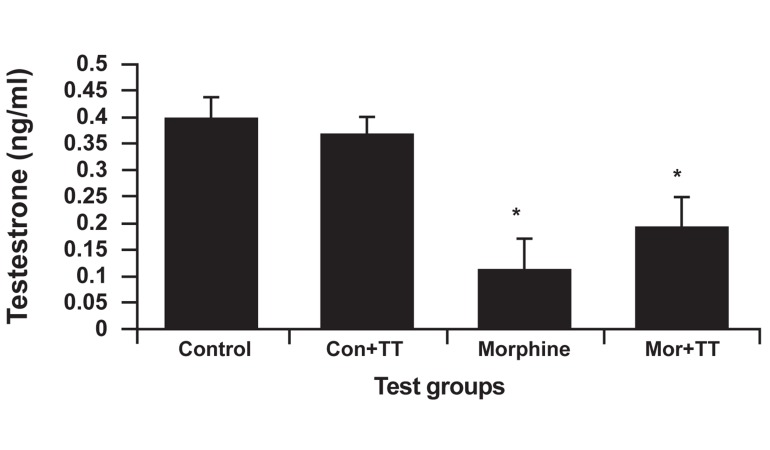
The effect of Tribulus terrestris (TT) on testosterone levels in control and addicted groups. Bars depict mean ± SEM of testosterone. *; P<0.05 compared between the control and treated control groups.

In this analysis, there was a reduction in the addicted group (0.122 ± 0.058 ng/ml). The highest amount of testosterone belonged to the control group (0.399 ± 0.04 ng/ml). Figure 3 shows that the addicted group had the least amount of testosterone, as a result of morphine suppression. The treated addicted group had a significantly lower hormone level (p<0.024) than did the treated control group (0.193 ± 0.057 ng/ml). The addicted group had a significantly lower testosterone level than did the control group (p<0.001).

### Estrogen analysis

**Fig 4:**
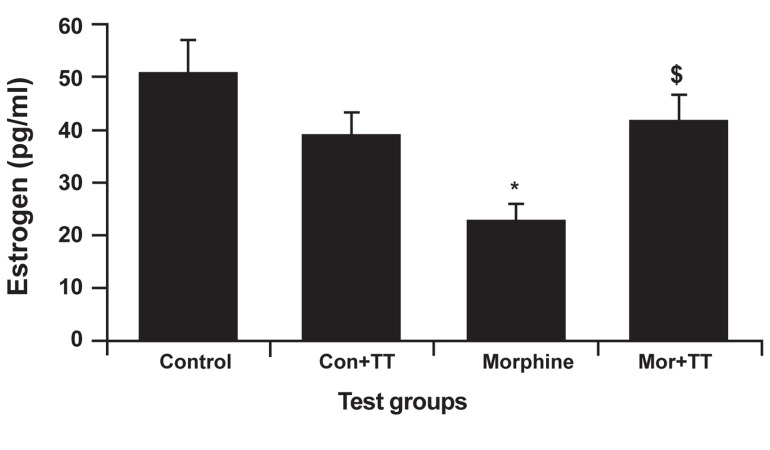
The effect of Tribulus terrestris (TT) on estrogen levels in control and addicted groups. Bars depict mean ± SEM of LH. * and $; P<0.05 compared between the control and treated control groups.

Our estrogen analysis revealed a decrease in the addicted group compared to the control group (22.70 ± 3.21 pg/ml). Figure 4 illustrates these results. Compared to the control group, estrogen decreased in the addicted group; there was a significant difference between these two groups (p<0.002). Figure 4 also shows a significant difference between the treated addicted group and the treated control group (p<0.048).

### Progesterone analysis

**Fig 5:**
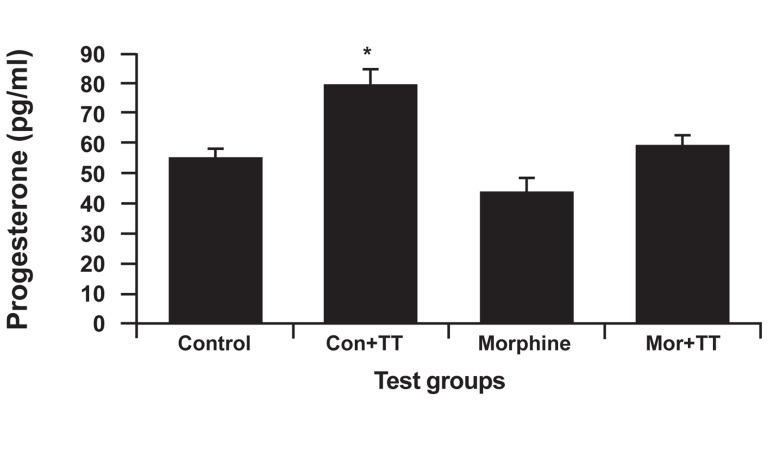
The effect of Tribulus terrestris (TT) on progesterone levels in the control and addicted groups. Bars depict mean ± SEM of progesterone. *; P<0.05 compared between the control and treated control groups.

There was an increase in the treated control group and a decrease in the addicted group according to progesterone analysis. Figure 5 shows that the treated control group had the highest hormone level (79.28 ± 5.2 pg/ml), which was due to TT. The addicted group had the least amount, which was due to the effects of morphine. The addicted group had a lower progesterone level than did the control group; the difference, however, was not significant. In comparison with the treated addicted group, the treated control group had a significant increase in hormone levels (p<0.002).

## Discussion

In this study, sex hormone and gonadotropin levels were evaluated based on the effect of TT on morphine-addicted rats. FSH, a stimulating hormone of the ovarian follicles, had a higher level in our morphine-addicted groups. In the treated morphine group, the FSH level had the most decrease, which demonstrated that TT suppressed FSH release in the addicted rats. A similar result was observed in the treated control group; however the difference was not significant.

Tabakova and his colleagues, in a comparison of endocrinal functions before and after TT therapy, reported that TT acted on the hypothalamus and reduced FSH levels but did not decrease ovarian hormones (estrogen and progesterone). They concluded that TT could be used to treat menopausal symptoms such as hot flashes and increase sex drive. These researchers speculated that the presence of saponin in TT was responsible for FSH suppression and the resultant alleviation of hot flashes, irritability, and depression in menopausal women (16). However, in our study, no significant effect of TT on FSH levels was found.

We observed a significant decrease in LH levels in the morphine-addicted group. The treated addicted group had a relatively higher LH than did the addicted group, which denoted that TT increased LH levels. Similarly, the treated control group had a significantly higher LH level than did the control group.

According to Neychev et al. (17) and a study by Antonio et al. it was shown that TT induced LH release. The upswing in LH led to a signal for testosterone to increase (18). With regard to testosterone levels, there was a significant decrease in our addicted rats. In the treated addicted group, the level was significantly higher than that of the addicted group. Although TT affected the increase in testosterone, a number of studies have demonstrated different results. Studies demonstrated that TT caused an increase in testosterone, increase in cavernous body pressure, increase in systolic pressure, and increase in penile erection (19-22). In addition, the effect of TT on castrated rats compared to normal rats was an increase in prostate weight, which augmented sexual activities and potency (22). In contrast, Neychev and his colleague reported that TT did not influence androgen levels in young men (17). Our results support the results of a majority of similar studies in the literature.

Based on our study, estrogen decreased significantly in the addicted group but increased significantly in the treated addicted group. It can, therefore, be concluded that TT significantly increased the estrogen hormone level in male addicted rats. Of note, there was a dearth of specific studies on estrogen in the literature.

Progesterone decreased in our addicted group; the amount, however, was insignificant. In contrast, progesterone significantly increased in the treated control group. We concluded that TT increased progesterone in non-addicted cases; however this increase was not significant in addicted cases. Mazaro-Costa and colleagues have reported that not only could TT treat sexual disorders and dysfunction in menopausal women but it could also enhance vasomotor actions. There are, however, no reports on progesterone specifically (19).

## Conclusion

The present study shows that addiction decreases sex hormones and gonadotropins. Treatment with TT can increase the hormone levels of testosterone, progesterone, estrogen, and LH. Nevertheless, TT did not increase the FSH levels in our male rats.
